# Student evaluations of teaching and the development of a comprehensive measure of teaching effectiveness for medical schools

**DOI:** 10.1186/s12909-022-03148-6

**Published:** 2022-02-19

**Authors:** Constantina Constantinou, Marjo Wijnen-Meijer

**Affiliations:** 1 University of Nicosia Medical School, Nicosia, Cyprus; 2 Technical University of Munich, School of Medicine, TUM Medical Education Center, Ismaninger Straße 22, 81675 Munich, Germany

**Keywords:** Student evaluations of teaching, Medical education, Course and faculty evaluation, Teaching effectiveness, Student learning

## Abstract

The evaluation of courses and faculty is of vital importance in all higher education institutions including medical schools. Student Evaluations of Teaching (SETs) commonly take the form of completion of anonymous questionnaires and even though they were originally developed to evaluate courses and programmes, throughout the years they have also been used to measure teaching effectiveness and subsequently to guide important decisions related to the faculty's career progression. Nevertheless, certain factors and biases may influence SET rates and may not measure teaching effectiveness objectively. Although the literature on course and faculty evaluations is well-researched in general higher education, there are concerns with regards to the use of the same tools for evaluation of courses and teachers in medical programmes. Specifically, the SETs in general higher education cannot be directly applied to the structure of courses and delivery of curriculum in medical schools. This review provides an overview of how SETs can be improved at the levels of instrumentation, administration and interpretation. In addition, the paper supports that through the collection and triangulation of data from multiple sources, including students, peers, program administrators and self-awareness via the use of different methods such as peer reviews, focus groups and self-evaluations, it will be possible to develop a comprehensive evaluation system that will present an effective measure of teaching effectiveness, will support the professional development of medical teachers and will improve the quality of teaching in medical education.

## Background

The assessment of courses and programmes is an internal process of quality control for all higher education institutions including medical schools. Student Evaluations of Teaching (SETs) commonly take the form of completion of anonymous paper based or online questionnaires using rating scales such as the Likert scale (frequently a five, seven or higher point scale) which allow the individual to express how much they agree or disagree with a particular statement) [1–3]. Even though SETs were originally developed to evaluate courses and programmes, throughout the years they have also been used to measure teaching effectiveness [4–6]. Teaching effectiveness is considered important because it is assumed that there is a positive correlation between the latter and student learning [7]. Although there is no clear definition of teaching effectiveness in the literature, it is usually operationalized by means of specific teaching characteristics such as “group interaction”, “preparation and organisation” and “feedback to students” [8].

The information derived from SETs may provide useful information such as the need to adapt the teaching material or the teaching method used in a particular course. SETs have also been used to guide important decisions related to the faculty's career progression [4–6]. However, it is questionable whether this method is suitable when higher education institutions make decisions about faculty, such as their promotion to higher academic ranks (which is commonly associated with tenure and salary increases) and significant administrative posts within the institution [4, 9]. In addition, institutions commonly require new faculty to include SETs from previous institutions in their applications for a new post, thereby affecting faculty’s progression not only within their institution but also with possible new employers [10].

Although the literature on course and instructor evaluations is well-researched in general higher education, this is not quite the case for the medical and health professions [11]. The needs of courses and faculty in medical education are different than those used in general higher education. For example, team teaching in often used in integrated curricula in medical education. This means that courses in medical schools consist of a series of classes run by multiple instructors with training and expertise in different medical subjects. Even though under this structure, students benefit from gaining in depth knowledge from specialists in the field, they are often challenged with adapting to the different teaching styles of each instructor [1, 12–14].

Despite the differences in general higher education compared to medical education, SETs used in the former are sometimes also used in medical and healthcare professional programs. However, there are numerous challenges in implementing SETs used in general higher education in the evaluation of courses and faculty of medical and healthcare professional programs [11]. Specifically, since there are differences in instructors’ teaching methods and proficiency levels, the results of course evaluations cannot include students’ perceptions of all instructors or classes. Research conducted by Uijtenhaage and O´Neal (2015) [5] shows that it may not be appropriate to ask students to evaluate all individual teachers at the end of a course since it is practically impossible for students to remember and comment on multiple instructors’ classes. Furthermore, many teachers in medical education are also physicians for whom teaching is a minor part of their responsibilities [15, 16]. Since they are primarily occupied with patient care, and in many cases research, there is often little time for development of their teaching skills. Nevertheless, physicians must be given time, support and constructive feedback by their organization in their role as teachers [16].

Medical students are usually highly motivated and hard-working individuals that managed to get admitted into medical school (through highly competitive and demanding processes internationally). Furthermore, during their time at the medical school, medical students are expected to acquire a great deal of knowledge and develop numerous skills in short periods of time and are expected to succeed in difficult internal and commonly national assessments [17–20]. Consequently, due to the high standards expected from them, medical students may be more critical and have higher expectations of what constitutes high quality teaching compared to students from other disciplines. It is therefore possible that due to the aforementioned reasons, medical students may provide lower evaluation scores of teachers compared to students from other disciplines. Interestingly, previous research has shown a positive association between student motivation and evaluation scores of individual teachers [21]. In addition, the curricula of most medical schools worldwide have become vertically integrated over the last 20 years [22] thereby students are exposed to clinical practice from the initial years of their programme. Consequently, in the past few years there is an increase in the involvement of physicians in the teaching of medical students even from the early years of their programmes supporting the importance of designing SETs to meet the characteristics of the particular group of teachers [22].

Due to the specific nature of medical education discussed above, SETs that are used to evaluate courses in general higher education that are delivered by one instructor have to be adapted to assess the integrated curriculum and clinical teachers of medical programmes [14]. There is therefore a need to develop a more effective SET model and a comprehensive evaluation system that could be applied more effectively in medical education.

The current review describes the recent developments on the use of SETs in (general) higher education and their limitations and subsequently provides an overview of the different needs of SETs for courses and faculty in medical education. The review provides an update of how SETs can be improved at the levels of instrumentation, administration and interpretation and focuses on the goal to develop an effective SET model and a comprehensive evaluation system that will present an effective measure of teaching effectiveness, will support the professional development of medical teachers and will improve the quality of teaching in medical education.

## Methods

The present study follows Green et al. (2006) [23] suggestions and Baumeister’s (2013) [24] recommendations on writing narrative reviews. We have chosen to write a narrative review on the topic since such a type of review is helpful in presenting a broad perspective on a topic. In addition, since narrative reviews draw on methodologically diverse studies, they help answer a broader number of questions. Moreover, narrative reviews serve to provoke thought and controversy on a topic.

The specific broad questions we wanted to ask were.

1. How are SETs currently used in general higher education and what are some of their limitations,
2. How are SETs used in medical education and what challenges have been reported compared to SETs used in general higher education,
3. How can SETs be improved at the levels of instrumentation, administration and interpretation

A search on Pubmed and ERIC databases was conducted using a combination of the search terms “Student Evaluations of Teaching” ,“Teaching Effectiveness” , “Medical Education, “Higher education”, “Course and Faculty Evaluation” and applying Boolean operators for peer reviewed journal articles published during the period of 2000-2021. Inclusion criteria: Included studies- were original studies or review articles examining areas falling under the three main research questions. Exclusion criteria: Non-English studies or studies for which full text articles could not be retrieved or were not related to the three main research questions were excluded from the current review paper. Once publications were selected they were organised under the following themes and their related sub-themes: (a) The use of SETs in general higher education and their limitations, (b) the use of SETs in medical education and associated challenges compared to SETs used in general higher education and (c) Improving SETs at the levels of instrumentation, administration and interpretation, with the aim to develop an effective SET model.

## Results

**Fig. 1:**
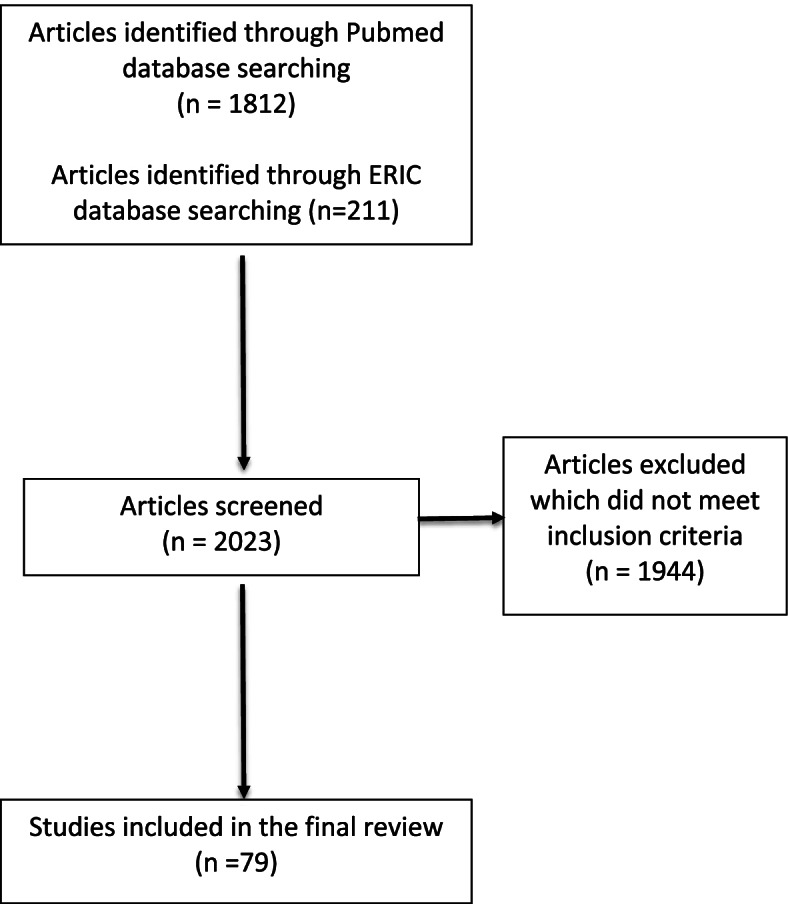
Flow diagram providing an overview of selected articles included in the current review article

Figure 1 provides a flow chart of the selected articles included and discussed in the current section of this review.

### The use of SETs in general higher education and their limitations

SETs have been used traditionally in higher education and this topic is very well researched in the literature [10, 21]. Yet, their numerous limitations as well as efforts to address them have been addressed by numerous studies.

**Fig. 2:**
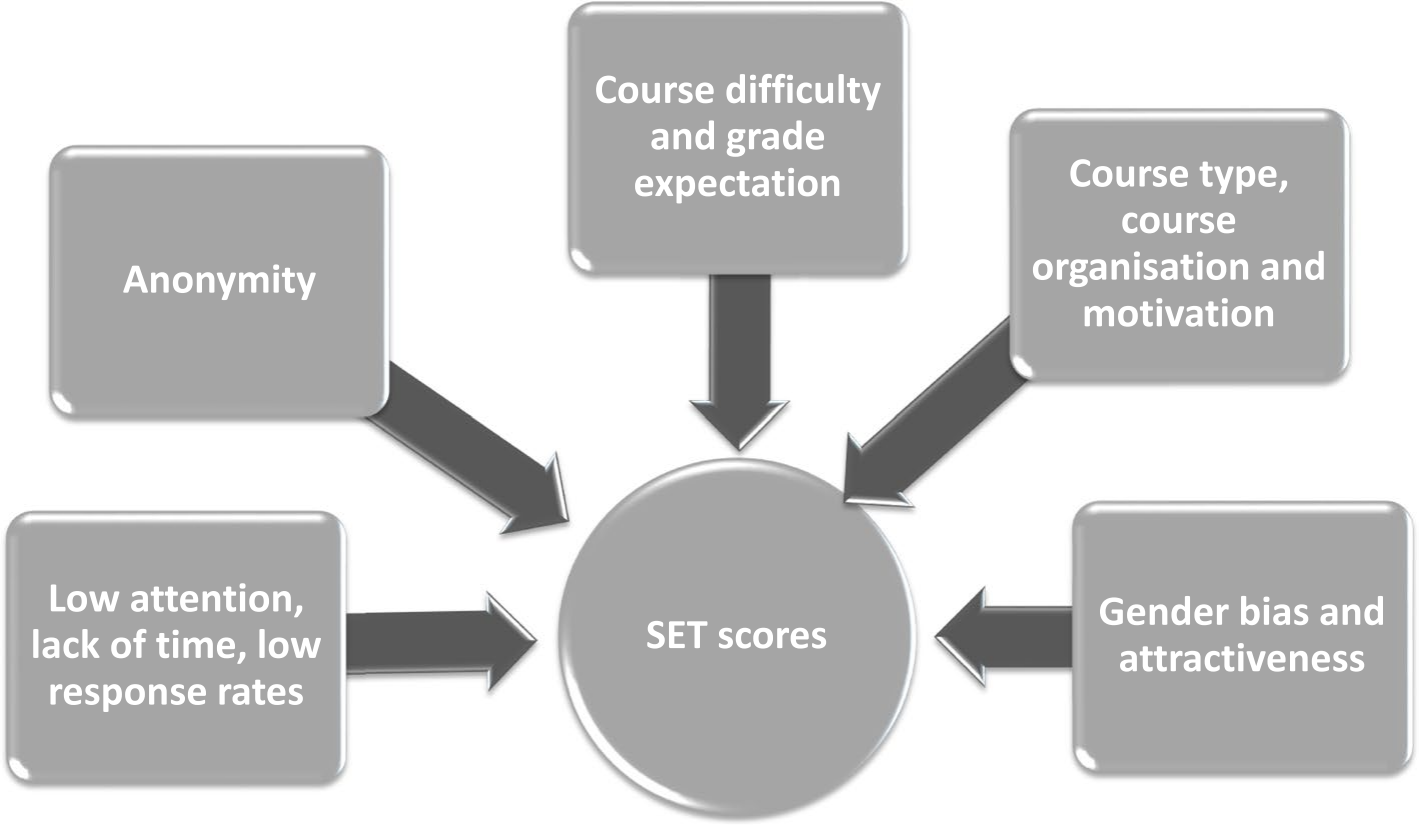
Factors that influence the scores of Student Evaluations of Teaching (SETs)

Research shows that there are a number of variables that influence SET scores [10, 21, 25, 26]. It is therefore important that administrators and faculty are aware of these variables when interpreting and using the data. The following sections provide a short overview of these variables. Figure 2 provides an overview of some of the factors that influence the scores of SETs which are further described in the section below.

#### Low attention, lack of time and low response rates

In the recent years, there has been an increase in the use of online compared to paper based SETs. However, evidence in the literature supports that online SETs may be completed without the necessary attention paid by the students during the completion process. In an interesting study by Uijtdehaage and O´Neal [5], non-existent faculty were added to the SET, to which many students gave feedback [5]. In addition, evidence in the literature supports that students often feel that completing SETs does not lead to an improvement in education and the latter combined with the busy schedule of medical students, can lead to a low response rate [27]. Although research shows that the opinion of the students who complete the evaluations does not deviate from the total group a low response rate can still lead to faculty taking the results less seriously [28].

#### Anonymity

Most of the online SETs are completed anonymously. The idea is to allow students to express their opinion freely and not feel that the expression of their opinion will have any consequences on their future relationship with faculty. In a study by Alfonso et al. [29], the researchers assessed teaching performance of medical school faculty by residents and medical students using both anonymous evaluations and evaluations in which the evaluators had to provide their names (open evaluations). The results of the study showed that overall faculty received lower scores in the anonymous evaluations. The authors believed that students were more honest in the anonymous evaluations due to certain barriers in the open evaluations such as the destruction of a working relationship with the faculty involved [29]. Nevertheless, it should also be noted that the anonymity commonly associated with the online SETs may allow some students to be disrespectful and vindictive towards faculty if for example the grade received in an assessment does not fulfil a student's expectations [30]. Nevertheless, research shows that it is not common for students to provide disrespectful feedback and the latter can be further limited by teaching students how to give constructive feedback [30].

#### Course difficulty and grade expectation

Several studies have shown a correlation between the students' SET scores, their expectations of the examination grade and their satisfaction with the exams [10, 21]. For example, Stroebe (2020) [9] have reported that students reward easy courses and teachers who grade leniently with positive SETs, and the latter may encourage poor teaching and contribute to grade inflation [9]. In a recent study by Looi et al. (2020) [31] the researchers reported that there was an association of more favourable SET for easier assessment. Furthermore, there is some concerning emerging evidence that SET is inversely proportional to the performance of students in subsequent courses, i.e., the higher the ratings, the poorer the students perform in subsequent studies. Kornell et al. (2016) [32] conducted a study to investigate whether university students learn relatively more from teachers whom they rate highly SETs. The results of the study indicated that when learning was measured at the completion of the course, the teachers who received the highest ratings also contributed to most of the student learning. However, when learning was measured as performance in subsequent related courses, the teachers who had received relatively low ratings appeared to have been most effective. The researchers speculated that making a course difficult in productive ways may decrease ratings but enhance learning. Therefore, student evaluation scores should not be the sole basis for evaluating teaching and they should be recognized for what they are.

#### Course type, course organisation and motivation

Several studies have provided evidence that the SET rates are affected by the actual course and its organisation. In their study**,** Min and Baozhi [33] found that there were significant differences among the SET rates of students on different subjects. For example, clinical sciences received higher SET scores than basic sciences. The authors explained that this was due to the fact that medical students were interested to become doctors so they had a personal interest and high motivation to engage more in the clinical science courses compared to the basic science courses [33]. The students' motivation for the subject, as is the case with electives, also has a positive effect on the scores [21]. Several other studies have also supported that the type of course may have an effect on the SET rate [10, 21].

In addition, other studies showed that the smaller the size of the classroom, the higher the SET rates received by faculty [10, 33]. A possible explanation is that a small classroom number allows an increased opportunity for faculty and student interaction. Furthermore, the conditions under which the evaluations are conducted also influence the results. For example, it appears that the SET scores are affected depending on the time and day a course is taught as well as which day of the week the SET is completed (e.g., evaluations completed at the end of the week often lead to more positive scores than evaluations completed earlier in the week [10].

The validity of SETs has been questioned also by an interesting study by Hessler et al. [34]. In this study, a randomised controlled trial was carried out in the setting of an emergency medicine course. Third-year medical students were randomly allocated either to a control group or a group in which they had free access to chocolate cookies (cookie group). All groups were taught by the same teachers and the educational content and course material was identical in both groups. After the course, all students were asked to complete an SET. The results showed that the cookie group evaluated the teachers significantly better than the control group thereby questioning the validity of SETs [34].

#### Gender bias and attractiveness

Evidence in the literature also supports that gender may influence SET scores [35–46]. For example, some studies that show a link between the gender of the students and the evaluation results, with female students giving higher scores when compared to male students [27]. Most of the evidence supports that students give female faculty lower evaluation scores than male faculty [37–40]. For example, Boring et al. [38], has shown that men are perceived by both male and female students as being more knowledgeable and having stronger leadership skills than women. The fact that gender and stereotyping affects SETs has also been supported by a study by MacNell et al. [41], who reported that in his study, students ranked female faculty lower than men on different aspects of teaching [41]. Furthermore, Morgan et al. [42] provided evidence that female physicians received lower teaching evaluations in four core clinical rotations (surgery, paediatrics, obstetrics and gynaecology and internal medicine) when compared to male physicians.

In a study by Murray et al. (2020) [43] the researchers reported that faculty attractiveness and the student's interest in the course were associated with higher SET rates. On the contrary, course difficulty was associated with lower SET scores. In addition, students gave higher SET scores to young, male, white faculty teaching in the humanities and holders of a full professor rank. No association was reported between SET scores for teaching and the faculty’s research output. Additional research also supports that physical attractiveness of faculty has a positive effect on the evaluation results [44].

#### Issues of reliability

Clayson et al. (2017) [45] reported that even though SETs are generally thought to produce reliable results and consistency is found within class and instructor averages, inconsistency exists with individual student responses. Therefore, the results of this evaluation report that students do not agree on what they are being asked to evaluate. The reliability measures generated by the student evaluations of teaching are an insufficient foundation for establishing validity. Consequently, SETs may sometimes be providing information about students, not instructors.

### The use of SETs in medical education and associated challenges compared to SETs used in general higher education

Medical education SETs differ from traditional SETs, yet faculty often use SETs available in general higher education as opposed to SETs reported in the literature to have been used specifically in medical and healthcare professional programs. However, issues have been reported by research conducted over the years.

Jones et al. (1994). [46], performed a study to identify problems in how medical school faculty are evaluated, from the perspectives of faculty and administrators. Overall, the most frequently mentioned problems were focused on the evaluation of teaching. General complaints about the inadequacy of current methods to evaluate teaching performance were most common and the respondents also had specific complaints about SETs and the insufficient recognition given to teaching in the academic reward system. Other concerns reported included the nonuniformity of evaluation processes and criteria for promotion among departments, the absence of regular evaluations, and the failure to link the results of evaluation to salary.

Royal et al. (2018) [11] have provided an overview of some of the limitations of using SETs used in general higher education for the evaluation of courses and faculty of medical and healthcare professional programs. The researchers report that a variety of different problems are faced since the SETs in higher education cannot be directly applied to the structure of courses and delivery of curriculum in medical schools. Common problems include the fact that questions about the instructor and the course are often combined onto a single questionnaire; thus, students often have difficulty differentiating between the two. Furthermore, courses in medical programs are typically taught by multiple teachers. This creates validity concerns given the number for potentially limited interactions between students and the instructors evaluated Royal et al. (2018) [11]. In a study by Hwang et al. (2017) [14], the researchers explored the notion of whether a retrospective course evaluation comprehensively captures student perception of classes by different instructors. The results of their study provided evidence that individual class evaluation is needed to manage multi-instructor courses in integrated curricula of medical schools.

Uijtdehaage and O’Neal (2015) [5] investigated the degree to which medical students complete SETs deliberately in a classroom-style, multi-instructor course. One fictitious lecturer was included into each of two pre-clinical courses. Students were required to submit their anonymous ratings of all lecturers, including the fictitious one, within 2 weeks after the course but could choose not to evaluate a lecturer. The following year, this was repeated but a portrait of the fictitious lecturer was included. Without a portrait, 66%of students evaluated the fictitious lecturer, but fewer students (49%) did so with a portrait. These findings suggest that many medical students complete SETs mindlessly, even when a photograph is included, without careful consideration of whom they are evaluating and much less of how that faculty member performed. This hampers programme quality improvement and may harm the academic advancement of faculty members. The researchers presented a framework that suggested a fundamentally different approach to SET that involves students prospectively and proactively.

A number of other differences also exist with respect to the educational curriculum of medical programmes compared to other programmes in general higher education [11]. Medical education as well as education in the healthcare professions uniquely focuses on the development of a defined professional role (clinical practice). Therefore, the curricula in medical and healthcare programmes are much more static, with limited options for course and instructor selection. Interestingly, courses in medical education are commonly delivered in a cohort format i.e., by concurrently enrolling all members of a class in the same courses each term. Therefore, the enrolment of larger numbers (often *n* = 100 or more) of students may affect teaching formats as well as relationships between faculty and students. In addition, in many medical schools, the psychometric properties of most instruments are not evaluated upon initial administration, and the properties for most instruments may remain unknown [11].

### Improving SETs at the levels of instrumentation, administration and interpretation, with the aim to develop an effective SET model

**Fig. 3:**
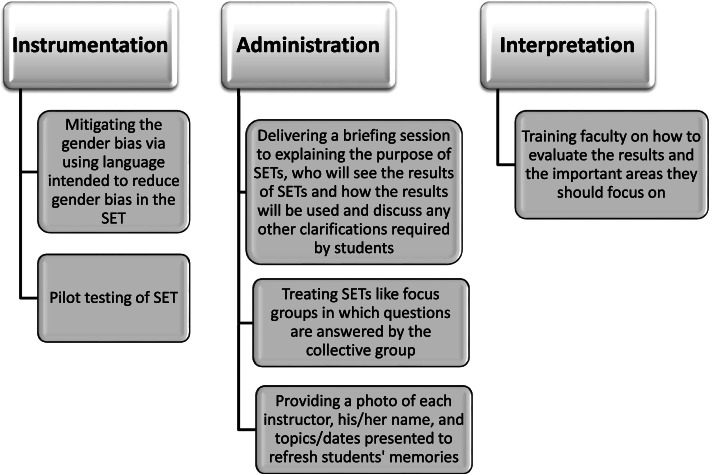
Improving SETs at the levels of instrumentation, administration and interpretation to develop an effective SET model

Several research studies conducted in the past few years have provided evidence that SETs could be improved by addressing some important factors that may influence the validity of SETs at the levels of instrumentation, administration and interpretation. Figure 3 provides an overview of some of the activities that could be incorporated to produce an effective SET model. A more detailed description is provided in the section below.

#### Instrumentation

##### ***Mitigating the gender bias***

As mentioned previously, the literature supports that gender bias may affect faculty evaluations [35–46]. Peterson et al. (2019) [40] conducted a study in which they examined if the sex of the student may shape how students respond to efforts to mitigate biases. In this study, SETs were conducted in four classes (two taught by male faculty and two taught by female faculty). In each of the courses, students were randomly assigned to either receive the standard evaluation instrument or the same instrument with language intended to reduce gender bias. The study reported that students that had used the anti-bias evaluation instrument had given significantly higher SET scores to female faculty compared to students that had used the standard evaluation tool. In addition, no differences were reported between the two groups for their evaluations of male faculty. The results of the study are significant and provide evidence on how a relatively simple intervention in language can potentially reduce gender bias in student evaluation of teaching. It is therefore good practice for all SETs to be carefully reviewed and to apply language intended to reduce gender bias during their development [40].

##### ***Pilot testing***

In order to obtain useful results from any SET, it is important that the purpose of the evaluation and the formulation of the questions are carefully considered in advance. While most SET surveys clearly indicate a section on the organisational aspects and content of the course i.e., ‘course evaluation’ and a section on faculty i.e., ‘faculty evaluation’, in some surveys the difference may not be clear or students may be confused as to how each of these areas should be evaluated separately. Therefore, the questionnaires should be designed appropriately so that the two different parts of the questionnaire are clearly indicated and students should be briefed regarding the content that should be assessed under each area. In addition, a pilot test is recommended, in which it can be determined whether the students interpret the questions in the way expected [24]. In a study by Oermann et al. (2018) [26], the researchers retrieved and summarised literature describing the use of SET across a broad range of disciplines in undergraduate and graduate education to provide guidelines for faculty in using SETs in nursing and other health professions programme. The results of the study reported that SET tools should be evaluated prior to their use including pilot testing these tools with students who may not interpret items or questions on an SET tool as faculty intended.

#### Administration

Several studies have investigated whether the mode of administration of an SET affects the students’ participation.

Dommeyer et al. (2004) [47] compared student evaluations of faculty teaching that were completed in-class with those collected online by comparing the response rates and evaluation scores. The results of the study showed that the response rate to the online survey was generally lower than that of the in-class survey. However, the study found that online evaluations did not produce significantly different mean evaluation scores than traditional in-class evaluations.

During the completion of online (but very often also hard copy) SETs, there has been a reported lack of bidirectional communication between students and faculty and therefore there is no opportunity for clarifications. Therefore, it may not always be clear what the questions are asking or what is meant by the students' comments or scores provided in SETs [48]. Several institutions have addressed this matter by gathering students for an hour and providing designated time to complete SETs online (anonymously) [49]. In their study Malone et al. (2018) [49] conducted sessions in which they discussed with the student the purpose of SETs, who will see the results of SETs and how the results will be used, as well as any other matters raised by the students. SETs were treated much like a focus group in which open-ended questions were answered by the collective group through informal polling, debate, and clarification. Response rates exceeded 70–80% thus providing faculty, administrators and curriculum committees substantial information [49].

As mentioned above, in a study by Uijtdehaage and O’Neal [5] the researchers reported that in their study, students provided ratings for non-existent instructors. As previously discussed, this is a common problem in medical school courses where each course may be delivered by many faculty members; yet students may not remember who contributed to each course and the quality of teaching exhibited by each faculty member. This problem has been addressed by some institutions by providing a photo of each instructor, his/her name and topics/ dates presented to refresh students’ memories and to avoid issues compromising the validity of the SETs [49].

#### Interpretation and closing the feedback loop

##### Training faculty in interpreting SET Results

Perhaps the most significant issue regarding SETs is that faculty continue to struggle with how to appropriately interpret the quantitative and qualitative results of SETs. Some faculty may want to do statistical comparison from year to year, some may want to attribute trivial gains/ losses to mean scores as meaningful changes, some want to believe every comment whereas others are entirely skeptical of any comments [45, 50, 51].

The inability to interpret results appropriately or to deal with student feedback may have an impact on how faculty feel about teaching. The results of Lutovak et al. (2017) [52] support that pedagogical training is necessary and useful in providing guidance for student feedback. Training for appropriately interpreting SET results is desperately needed in medical education. Therefore faculty in medical schools should be trained on how to evaluate the results and the important areas they should focus on [50, 51].

The results of the studies described therefore support that SETs should be carefully designed, administered and interpreted in order for the results of the SET to have a meaningful impact to all stakeholders involved including faculty members, medical school administration and students.

### Developing a comprehensive measure of teaching effectiveness

Due to some of the limitations associated with SETs, further efforts should be made to move towards a comprehensive evaluation system that will present a less biased measure of teaching effectiveness and will support the professional development of medical teachers.

**Fig. 4:**
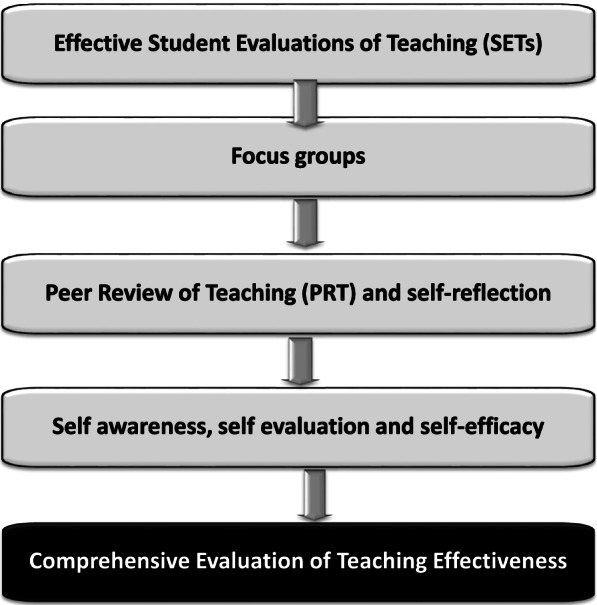
Methods that can be used in the development of a model for a comprehensive evaluation system for measuring teaching effectiveness in medical schools

A more comprehensive picture of clinical faculty teaching quality can be achieved through the collection and triangulation of data from multiple sources, including students, peers, program administrators and self-evaluation by faculty [53–57]. The following section describes possible additional instruments/ methods that could be used in addition to effective SETs to help the development of a more appropriate and holistic view of teaching effectiveness (Fig. 4).

#### Focus Groups

Focus groups are defined as “group discussions organized to explore a specific set of issues” [58]. In the past few years medical schools have implemented focus groups to support the extraction of qualitative feedback from students and to address some of the pitfalls for online SETs. These studies have supported that focus groups are effective in providing qualitative feedback and in increasing student satisfaction [59–61].

In a study by Brandl et al. [59], the researchers implemented a student evaluation team process that allowed course directors and students to discuss a course in a focus group. The results of the study showed that the focus group discussions were valuable in supplementing online evaluations and increased the students’ satisfaction with the overall course evaluation process. The students appreciated the opportunity to communicate directly with course directors and believed this process could support educational improvements. They also felt that they could understand the course director’s perspective. In addition to students, course directors also appreciated that the focus groups supported more effective communication with the students [59]. Therefore, using focus groups may give medical schools a more complete picture of the quality of each course and teaching effectiveness of the faculty involved. It should be noted however that focus groups on their own do suffer from some limitations such as the fact that only a small number of students participate in these compared to the online SETs which are accessible by all students. In addition, running focus groups for the different courses can be a time consuming process for both the facilitators as well as the students. This imposes significant restrictions especially for medical students that have very busy schedules and may be present in different geographical locations for their clinical placements. In addition, focus groups require having a significant number of highly experienced facilitators. Nevertheless, the inclusion of focus groups in the evaluation process provides more detailed and specific information on teaching effectiveness [48, 59–61].

Schikierka-Schwake et al. (2018) [62] investigated student and teacher perceptions of a novel evaluation tool assessing teacher performance and student learning outcomes in two German medical schools. Focus group discussions as well as one-to-one interviews involving teachers and undergraduate medical students were conducted. Teachers appreciated the individual feedback provided by the evaluation tool while students reported that in order to be motivated to provide evaluation data, feedback loops including aims and consequences should be established. Therefore, the results of this study support the importance of closing the loop with the students and informing them of the outcome of their evaluations.

#### Peer Review of Teaching and Self-reflection

Peer Review of Teaching (PRT) programmes are quite important and have been implemented in higher education for years. PRT involves the collaborative process of observation of teaching and the provision of feedback to the observee to improve teaching effectiveness [63]. In addition, self-reflection exercise, structured follow-up discussion and systemic assignment of trained peers can contribute to the effectiveness of PRT and to the teaching culture of the department [64]. These programmes have been reported to have many benefits since they help faculty receive constructive feedback from peer faculty who may have faced similar difficulties in the past and may be quite supportive in providing useful advice for improvement [63]. In addition, when peer reviews are used in a constructive way, they can improve course content and delivery and support medical teachers to improve the quality of their teaching [65, 66].

A recent study from Campbell et al. (2019) [67] provided evidence that a workplace-based peer support model is an acceptable and effective faculty development strategy for health care clinical teachers. In another study, Caygill et al. [68] carried out a study in which a purposefully designed questionnaire was sent to medical educators at the University of Melbourne to share their experiences on PRT. The results of the study showed there was a significant unmet interest among medical educators for PRT and that a voluntary and informative form of peer review was considered as an important and valuable opportunity for professional development.

It is important to mention that PRT programmes need to be carefully designed in order to avoid the creation of a judgmental, ‘managerialist’ environment which commonly leads to increased anxiety by the faculty being observed [69]**.** The aim should therefore be to carefully design a PRT programme that would supplement and contribute towards the development of a safe environment and provision of constructive feedback. Therefore, specific training should be put in place to train the peer reviewers and only truly interested and experienced faculty should be used in the PRT programme. This is particularly important if the information derived from PRTs is used for decisions about the faculty such as their promotions to higher ranks, salary increases and promotion to important administrative posts. It should be noted that PRTs are time consuming and similar to the focus groups require commitment from a significant number of experienced faculty thereby making this a difficult method to be implemented in medical schools with scarcity of resources.

Newman et al. (2019) [70] described strategies to use before, during, and after teaching observations that highlight best practices and identify solutions for teaching challenges. The researchers provided 12 tips to reviewers which included: (1) choosing their words wisely; (2) letting the observee determine the direction of the discussion; (3) keeping feedback confidential and formative (4) focus feedback on teaching skills, not the teacher as a person; (5) get to know their colleague (6) be aware of self and other (7) be aware pronouns play a powerful role in providing feedback, (8) use questions to uncover teaching perspectives, (10) establish credibility in the peer observation and feedback process, (11) make teaching observations a win win and (12) conclude with an action plan. The researchers also explored the impact of biases on observations, and how to make the teaching observation process and feedback discussion valuable experiences for both parties so that it leads to long-lasting partnerships and improvement in educational quality. Gormally et al. (2014) [71] have reported that the qualities of effective feedback should include (1) clarification of the task by providing instruction, (2) improvement of motivation to prompt increased effort and (3) the perception by the recipient as a valuable process because it is provided by a reputable source.

Even though faculty at medical schools receive feedback from PRTs, it is important to train faculty on how to interpret feedback (in a similar way as it has been proposed that they are trained to interpret SETs) and allow faculty enough time to reflect on the feedback received in a constructive manner.

There is evidence in the literature that reflection deepens the faculty’s understanding of their roles as educators and supports the development of a learner centred approach to learning [72, 73]**.** In their study, Winchester and Winchester [74] investigated the impact of reported reflective practice using SETs. Data were collected in a rural UK based university-college in 11 modules over a period of 2 years of data collection. Findings showed that on average, SET scores increased for all reflective practitioners and increased more for those faculty who demonstrated higher levels of reflection. Therefore, every faculty evaluation process should be linked to reflective practice thereby allowing faculty time to think and reflect on performance of a previous academic year and develop some goals for future professional development [72–74].

In a recent study, Bajwa et al. (2020) [75] conducted a study to investigate how junior clinical faculty can receive institutional support in the acquisition of feedback and clinical supervision skills of trainees. In the study the researchers tested the effectiveness of a personalised coaching versus guided self-reflection at improving faculty skills and self-efficacy. The results of the study showed that offering a faculty development programme using Objective Structured Teaching Exercises (OSTEs) that provided opportunities for feedback and focused on creating a community of practice, was effective in the transfer of skills to the clinical environment and supported teacher development. Furthermore, Carlson et al. (2020) [76] implemented and evaluated a faculty development program involving peer observation and feedback for attending physicians. The results of the study reported that the inpatient-based peer-coaching faculty development program was acceptable and feasible for a majority of faculty and may improve individual teaching effectiveness among conventionally trained physicians.

### Individual interviews?

In addition to the previously mentioned instruments, individual interviews with students also stimulate the reflection of faculty [75]. A study by Hoban and Hastings (2006) [77] showed that teachers benefit most from student feedback during personal interviews; yet this method is very time-consuming, both for teachers and students who may find it difficult to give feedback to teachers in a direct dialogue.

### Self-awareness, self-evaluation and self-efficacy

Self-awareness was measured in higher education in the past; yet it was not specifically used in relation to ‘effectiveness of teaching’. Gul et al. (2021) [78] developed and validated an instrument for medical teachers to measure the self-awareness of their teaching. The 19-item final instrument had four themes, that is; self-reflection, communication with students, student feedback, and peer review. The Self-awareness of the teaching skills instrument had excellent validity and good reliability in measuring the self-awareness of teaching skills of medical teachers [76].

Vaughan (2020) [57] conducted a study in which clinical educators in an osteopathy program at Victoria University (VU) were invited to complete: a) self-evaluation version of the Osteopathy Clinical Teaching Questionnaire (OCTQ) and b) the Self-Efficacy in Clinical Teaching (SECT) questionnaire. The completed questionnaires were matched with student evaluations. The researchers concluded that professional development directed towards developing self-efficacy may engage faculty whose self-efficacy is low and/or those who received low student evaluations. Vaughn et al. [57] reported that there is no gold standard at the present time to measure clinical teaching quality, and therefore teachers in medical education should engage with multiple sources of feedback to assess their performance, and identify opportunities to improve. Student and self-evaluations using the OCTQ and evaluation of self-efficacy using the SECT, seemed to be useful tools for inclusion in a comprehensive approach to evaluation in medical education.

Overall, the results of reflection and the personal goals should always be discussed with peers and faculty in higher academic ranks and administrative positions in the context of further development.

## Discussion

The literature supports that SETs should be carefully designed for the evaluation of teaching effectiveness in medical schools. Although the literature on course and faculty evaluations is well-researched in general higher education, this is not quite the case for the evaluation of medical courses [11].

Our narrative review attempted to address some broad questions related to SETs and their application in medical education. The paper addressed important questions and attempted to review the topic by trying to inform the reader about our current understanding on (a) the use of SETs in general higher education and their limitations, (b) the use of SETs in medical education and associated challenges compared to SETs used in general higher education. In addition the paper has explored how improving SETs at the levels of instrumentation, administration and interpretation could contribute towards the development of an effective SET model.

Our review has provided insight that when using SETs, it is important for administrators and faculty to be aware of important factors which may influence the results and which include among others low attention and lack of time from students, biases due to course difficulty and grade expectation, students’ personal motivation as well as gender biases [10, 21, 23].

Each SET should therefore be carefully designed to and reviewed to address the factors that affect SET scores. Efforts should be put in place at the level of instrumentation, administration and interpretation to design an effective SET that will evaluate course quality and teaching effectiveness in as an objective manner as possible. At the level of instrumentation, for example medical schools should try and use language to reduce gender bias and each new SET should be pilot tested prior to implementation. At the level of administration of a SET, a briefing session could be offered prior to the completion of each SET to answer any questions raised by the students. In addition, it may be useful that each SET is treated as a focus group by which questions are answered following a discussion between the group. Special efforts should also be made to ensure that students’ memories are refreshed for evaluating faculty that may not have significant contributions in the course. For example, photos and list of sessions delivered could be shown next to the names or each faculty member of short evaluations could be conducted after the completion of session when the students’ memory is fresh and their answers to the SETs may be more accurate of their true impressions. One of the obstacles commonly faced in SETs is that both faculty and students have different perceptions and expectations with regards to the scope and objectives of SETs. This initial obstacle should be addressed by sufficient preparation of both faculty (e.g., via specific training on objectives and interpretation of results of SETs) and also students (e.g., on objectives, how the results will be used and also by sharing how feedback from completed SETs has been addressed and implemented).

In addition to the use of more effective SETs, medical schools should aim to develop a comprehensive evaluation system that will present an effective measure of teaching effectiveness, will support the professional development of medical teachers and will improve the quality of teaching in medical education. The evidence from the literature supports that some of the methods that could be used include focus groups [59–61], peer reviews of teaching and self-reflection [63–65] and evaluation of self-awareness, self-evaluation and self-efficacy [57, 78]. The implementation of such a comprehensive programme should aim to collect and triangulate data from multiple sources, including students, peers, program administrators and faculty themselves and should aim to support the professional development of medical teachers and to improve the quality of teaching in medical education. One limitation of such a programme however, may be that a significant amount of effort and resources will be required by both faculty, students and administration to run such a programme and with possible increased burden for all involved. This possible limitation should be taken into consideration in the design of the programme which should be pilot tested prior to implementation. In addition, there should be careful consideration with regards to how the results from different methods of data collection could be combined and analysed and how the feedback should be provided to faculty and administrators.

In summary, the results of the study propose that all medical schools should constantly strive to improve the medical education offered to their students but for this to be achieved they should provide an encouraging and caring environment for their faculty. By developing a holistic evaluation system, the data collected will be more objective, the feedback provided to the faculty will be more accurate, and the mechanisms needed to support the faculty will be put in place to help them achieve their maximum teaching potential.

Finally, we understand that our study is a narrative review and therefore one of its limitations is that in our effort to answer a number of broad questions related to our topic we may have limited our capacity to focus in more depth in studies conducted in the related fields. It is therefore important that the main themes derived are revisited in more depth and primary research is conducted to derive more specific results to the questions raised.

## Conclusions

Despite placing a significant amount of effort, time and resources on SETs, the literature supports higher academic institutions including medical schools should not use SETs as the only method to measure teaching effectiveness. SETs should not be used solely by medical school administration to assess the quality of courses or to guide decisions about their faculty (such as their promotions to higher ranks, salary increases and promotion to significant administrative posts). Medical schools could still use SETs but these should be carefully designed, administered and interpreted based on the latest evidence from the literature and by involving students, faculty and medical school administration in this process [24, 28]. The ultimate goal should be for medical schools to develop an evidenced based comprehensive evaluation system. The evidence from the literature supports that some of the methods to be used include focus groups, peer reviews of teaching and self-reflection and self-awareness, self-evaluation and self-efficacy. By developing a holistic evaluation system, medical schools will support the professional development of medical teachers and will improve the quality of teaching in medical education.

## Data Availability

Not applicable.

## Abbreviation

SETs: Student Evaluations of Teaching
